# Supplementary material for: Polyketide synthase gene clusters in the ericoid mycorrhizal fungus Oidiodendron maius: genome-wide prediction, expression profiling, and functional characterization of knockout mutants
Source: Front Plant Sci. 2026 Sep 16;17:1924430. doi: 10.3389/fpls.2026.1924430 (PMC13623903; doi:10.3389/fpls.2026.1924430)
Supplement: Supplementary file 1 [file SupplementaryFile1.zip › Supplementary Materials/Supplementary Folder 1.zip!/Oidma1_AssemblyScaffolds_Repeatmasked/knownclusterblast/cluster46/jgi.p_Oidma1_135336_mibig_hits.html]

| MIBiG Protein | Description | MIBiG Cluster | MiBiG Product | % ID | % Coverage | BLAST Score | E-value |
| --- | --- | --- | --- | --- | --- | --- | --- |
| EPE34336.1 | Cytochrome\_P450 | BGC0001035 | Pneumocandin\_biosynthetic\_gene\_cluster | 29 | 94.465648855 | 219 | 1e-56 |
| AFT91399.1 | HtyF | BGC0000338\_c2 | Echinocandin\_B\_biosynthetic\_gene\_cluster | 29 | 95.6106870229 | 207 | 3.2e-53 |
| AMM63168.1 | AniF2 | BGC0001371 | Echinocandin\_B\_biosynthetic\_gene\_cluster | 27 | 94.2748091603 | 193 | 4.7e-49 |
| AAK11528.1 | PaxP | BGC0001082 | Paxilline\_biosynthetic\_gene\_cluster | 27 | 99.6183206107 | 179 | 9.2e-45 |
| BAM84045.1 | cytochrome\_P450\_monooxygenase | BGC0001260 | Terpendole\_E\_biosynthetic\_gene\_cluster | 32 | 71.9465648855 | 178 | 2.1e-44 |
| EAU36745.1 | hypothetical\_protein | BGC0000292 | Acetylaranotin\_biosynthetic\_gene\_cluster | 30 | 89.3129770992 | 173 | 5.1e-43 |
| CBF87243.1 | cytochrome\_P450,\_putative\_(Eurofung) | BGC0000680\_c2 | Austinol/dehydroaustinol,\_locus\_1\_biosynthetic\_gene\_cluster | 31 | 75.3816793893 | 160 | 5.8e-39 |
| EAU29532.1 | conserved\_hypothetical\_protein | BGC0000682 | Terretonin\_biosynthetic\_gene\_cluster | 29 | 74.8091603053 | 150 | 6e-36 |
| AAW88512.1 | P450\_monooxygenase | BGC0001081 | Lolitrem\_biosynthetic\_gene\_cluster | 32 | 65.0763358779 | 140 | 4.7e-33 |
| AMM63169.1 | AniH | BGC0001371 | Echinocandin\_B\_biosynthetic\_gene\_cluster | 26 | 89.5038167939 | 140 | 6.2e-33 |
| AFT91389.1 | EcdH | BGC0000338 | Echinocandin\_B\_biosynthetic\_gene\_cluster | 26 | 89.5038167939 | 136 | 8.9e-32 |
| EAL94100.1 | cytochrome\_P450\_monooxygenase,\_putative | BGC0000811 | Fumigaclavine\_C\_biosynthetic\_gene\_cluster | 30 | 72.9007633588 | 131 | 3.8e-30 |
| BAC20565.1 | cytochrome\_P450 | BGC0000039 | Compactin\_biosynthetic\_gene\_cluster | 27 | 90.6488549618 | 128 | 1.9e-29 |
| BAM84048.1 | cytochrome\_P450\_monooxygenase | BGC0001260 | Terpendole\_E\_biosynthetic\_gene\_cluster | 28 | 65.2671755725 | 127 | 5.4e-29 |
| CAP07651.1 | P450\_monooxygenase | BGC0001269\_c5 | Gibberellin\_A3\_/\_gibberellin\_A1\_/\_gibberellin\_A7\_/\_gibberellin\_A4\_/ | 30 | 73.4732824427 | 125 | 1.6e-28 |
| CAA75565.1 | cytochrome\_P450\_monooxygenase | BGC0001270\_c4 | Gibberellin\_A3\_/\_gibberellin\_A1\_/\_gibberellin\_A7\_/\_gibberellin\_A4\_/ | 28 | 76.5267175573 | 123 | 7.8e-28 |
| ABF20222.1 | P450\_monooxygenase | BGC0001081\_c2 | Lolitrem\_biosynthetic\_gene\_cluster | 28 | 77.2900763359 | 120 | 5.1e-27 |
| EAW09122.1 | cytochrome\_P450 | BGC0000983 | Cytochalasin\_biosynthetic\_gene\_cluster | 26 | 85.1145038168 | 120 | 6.6e-27 |
| ABA02241.1 | P450\_monooxygenase | BGC0000098 | Monacolin\_K\_biosynthetic\_gene\_cluster | 26 | 93.5114503817 | 115 | 2.1e-25 |
| CAP07653.1 | P450\_monooxygenase | BGC0001269\_c3 | Gibberellin\_A3\_/\_gibberellin\_A1\_/\_gibberellin\_A7\_/\_gibberellin\_A4\_/ | 27 | 76.9083969466 | 114 | 4.8e-25 |
| EAW09118.1 | cytochrome\_P450\_oxidoreductase\_GliF | BGC0000983 | Cytochalasin\_biosynthetic\_gene\_cluster | 28 | 67.3664122137 | 106 | 9.9e-23 |
